# Prediction of the Compressive Strength of Tailings-Based Cement Material Using Machine Learning Models with Experimental Validation

**DOI:** 10.3390/ma19122557

**Published:** 2026-06-12

**Authors:** Zhanming Zhong, Senrui Deng, Tao Liu, Xiuxin Li, Xin Ye, Weijun Yang, Jianyu Yang

**Affiliations:** 1 School of Civil and Environmental Engineering, Changsha University of Science & Technology, Changsha 410114, China; jianyuy@csust.edu.cn (Z.Z.); dengsenrui@163.com (S.D.); yxx@stu.csust.edu.cn (X.Y.); bangbanghq@163.com (W.Y.); 2 School of Civil Engineering, Guilin University of Technology, Guilin 541004, China; 2120230888@glut.edu.cn; 3 Shenzhen Raw Water Co., Ltd., Shenzhen 518000, China; 17625480085@163.com; 4 School of Hydraulic and Ocean Engineering, Changsha University of Science & Technology, Changsha 410114, China

**Keywords:** tailings-based cement materials, compressive strength, machine learning, SHAP analysis, experimental verification

## Abstract

Partially replacing cement with mine tailings offers a sustainable strategy for solid waste resource utilization. As a cement admixture, the compressive strength of tailings-based cement materials serves as a critical performance indicator. Machine learning (ML) techniques offer high efficiency, cost-effectiveness, and superior predictive accuracy. However, variations in the chemical composition of tailings often introduce uncertainties into model predictions. Consequently, this study developed an integrated approach incorporating chemical composition and activation methods as input parameters. Four optimized ML models were deployed to predict the compressive strength of tailings-based cementitious materials. Multiple metrics were employed to evaluate model performance, which identified the PSO-XGBoost model as the superior predictive architecture. SHAP analysis revealed that mechanical grinding, NaOH concentration, and the proportions of gypsum and tailings were the primary features influencing compressive strength. Experimental validation yielded a low prediction error of 8.7%, confirming the model’s high predictive accuracy. This research establishes a robust framework for predicting the strength of tailings-based cementitious materials, providing a theoretical foundation for solid waste upcycling.

## 1. Introduction

Mine tailings constitute a major category of industrial solid waste. Intensive mineral extraction leads to the continuous accumulation of these wastes, posing substantial economic and environmental challenges. Large-scale storage not only impedes the recovery of valuable components but also causes environmental degradation, including land encroachment, soil degradation, and heavy metal contamination [1,2,3]. Consequently, optimizing the recycling and valorization of tailings has emerged as a critical research priority.

Given the chemical similarities between tailings and cement, recent research has focused on the partial replacement of cement with properly activated tailings as supplementary cementitious materials (SCMs) for concrete production [4,5,6,7]. This strategy offers a scalable, high-value waste management solution while significantly reducing the carbon footprint of cement manufacturing. Previous studies have demonstrated that incorporating activated tailings inherently influences the performance of cement-based materials. In particular, compressive strength serves as the primary metric for assessing these tailings-based cementitious materials [8,9,10,11], typically evaluated using specimens after 28 days of standard curing. This key property is dictated by the activation method employed, with common techniques including mechanical grinding, alkaline activation, and sulfate activation. However, variations in the chemical composition and dosage of tailings often lead to discrepancies among reported strength values. For instance, Geng [12] reported that the activity of mechanically activated tungsten tailings depends on the replacement ratio, peaking at 20%. Meanwhile, Li [13] emphasized chemical composition as the decisive factor controlling binder performance. Specifically through the optimization of the raw materials’ calcium-to-silicon (Ca/Si) ratio. Notably, the activity of tailings is governed by multiple coupled factors rather than a single variable. For example, Saedi et al. [14] successfully replaced a portion of cement by applying a mechanochemical activation method, demonstrating that tailings activity is simultaneously affected by dosage and the specific activation mode. Although these studies elucidate the independent effects of individual factors, the mechanisms underlying their synergistic interactions remain poorly understood. Consequently, a systematic investigation into these coupling effects is essential to establish a robust theoretical framework for solid waste upcycling.

Recent advances in artificial intelligence have established machine learning (ML) as a powerful tool for predicting the properties of tailings-based cementitious materials [15,16,17], with algorithms such as BPNN, RF, SVM, and XGBoost being widely adopted due to their predictive accuracy and robustness. To further enhance prediction accuracy and generalization, metaheuristic optimization algorithms have been integrated to automate hyperparameter tuning, thereby preventing premature convergence to local optima and enhancing model stability [18,19]. For instance, Alyami et al. [20] hybridized the firefly algorithm (FFA) with SVM to elevate strength predictions for copper tailings concrete, while Sobuz et al. [21] utilized grid search to optimize XGBoost, achieving high-precision predictions for nanomaterial concrete composites. Similarly, Wang et al. [22] and Miao et al. [23] applied Particle Swarm Optimization (PSO) and Bayesian optimization, respectively, to refine BPNN and XGBoost architectures, thereby improving the prediction of concrete compressive strength. While the application of ML frameworks to predict tailings-based material performance is rapidly evolving, hybrid machine learning models systematically achieve superior predictive accuracy compared to standalone baseline models [24,25,26,27].

Although machine learning has been widely implemented to predict the properties of tailings-based concrete, knowledge gaps persist regarding the fundamental behavior of tailings within cement-based systems. To bridge these gaps, this study integrates multidimensional variables—including chemical composition, mechanical grinding, alkali/sulfate activation, and tailings dosage—to develop an optimized ML framework. This model quantifies the synergistic effects of these coupled factors on the compressive strength of the resulting tailings-based cementitious materials. Furthermore, Shapley Additive Explanations (SHAP) analysis is deployed to identify the critical features dictating strength development, the individual relationships of which are further elucidated through partial dependence plots (PDPs). Finally, experimental validation using tungsten mine tailings is conducted to corroborate the predictive accuracy of the optimized framework, the findings of which provide valuable insights and practical guidance for the sustainable upcycling of industrial solid waste.

## 2. Research Methods

### 2.1. Machine Learning Algorithms

#### 2.1.1. The Principle of Limit Gradient Boosting (Xgboost)

XGBoost [28] demonstrates superior performance in both regression and classification tasks. The algorithm accelerates optimization by employing a second-order Taylor expansion of the loss function and incorporating a regularization term that penalizes model complexity, thereby mitigating overfitting. The objective function is defined as shown in Equation (1):

(1) $$obj(\theta )=\sum_{i=1}^{n}L(y_{i},\hat{y_{i}})+\sum_{j=1}^{m}\Omega (f_{j})$$

where n is the total number of samples, m is the number of decision trees, $L(y_{i},\hat{y_{i}})$ denotes the loss function measuring the discrepancy between the experimental value $y_{i}$ and the predicted value $\hat{y_{i}}$, and $\Omega (f_{j})$ represents the regularization term used to penalize the complexity of the j-th tree to prevent overfitting.

#### 2.1.2. Random Forest (Rf)

Random Forest (RF) [29] is an ensemble learning algorithm built on the architecture of decision trees (DTs). An RF model aggregates an ensemble of individual decision trees to improve predictive stability. Each tree is trained on a bootstrap sample of the dataset and generates independent predictions. The final output is determined through a collective voting or averaging mechanism. The predictive performance of the RF model is primarily governed by hyperparameters such as the number of trees and the maximum depth of each tree.

#### 2.1.3. Support Vector Regression (Svr)

Support Vector Regression (SVR) [30] aims to find a function f(x) such that the prediction error for all samples lies within an ∈-insensitive tube. Slack variables $\xi_{i}$ and $\xi_{i}^{*}$ are introduced to allow a certain degree of error. The optimization problem of SVR is shown in Equation (2):

(2) $$\min\frac{1}{2}{\left\|w\right\|}^{2}+C\sum_{i=1}^{n}(\xi_{i}+\xi_{i}^{*})$$

In order to deal with the nonlinear relationship, SVR introduces the kernel function K and maps the data to the high-dimensional space. The final regression function is shown in Equation (3):

(3) $$f(x)=\sum_{i=1}^{n}(a_{i}-a_{i}^{*})K(xi,x)+b$$

where $\alpha_{i}$ and $\alpha_{i}^{*}$ are Lagrange multipliers.

#### 2.1.4. Back Propagation Neural Network (Bpnn)

The Back Propagation Neural Network (BPNN) [31] is a fundamental multilayer feedforward architecture. Model training consists of two primary phases. First, input signals undergo forward propagation through successive layers of weights, biases, and activation functions to generate the predicted output. Then, the error between the predicted output and the true label is calculated, and the backpropagation algorithm is employed to iteratively update network parameters in reverse order, thereby minimizing the loss function and optimizing model performance. The mathematical framework for parameter updates is given by Equation (4):

(4) $$y_{i}=\sum_{j=1}^{n}(w_{ij}x_{j})+b_{i}$$

where $y_{i}$ is the output of the neuron, $x_{j}$ is the input from the preceding layer, $w_{ij}$ is the weight, and $b_{i}$ is the corresponding bias term.

### 2.2. Particle Swarm Optimization (PSO)

Particle Swarm Optimization (PSO) [32] is a metaheuristic algorithm inspired by the social foraging behavior of bird flocks. Within this framework, each candidate solution is conceptualized as a “particle” characterized by position and velocity vectors in the multidimensional search space. During the iterative process, particles dynamically adjust their trajectories by tracking both their individual best position and the swarm’s global best position, thereby facilitating progressive convergence toward the global optimum. Owing to its algorithmic simplicity, rapid convergence, and robust global search capabilities, PSO is widely used for hyperparameter optimization in machine learning. The velocity and position update mechanisms are mathematically expressed in Equations (5) and (6):

(5) $$v_{i}^{t}=wv_{i}^{t-1}+c_{1}r_{1}(pbest_{i}^{t}-x_{i}^{t})+c_{2}r_{2}(gbest^{t}-x_{i}^{t})$$

(6) $$x_{i}^{t+1}=x_{i}^{t}+v_{i}^{t}$$

$v_{i}^{t}$ and $x_{i}^{t}$ signify the velocity and position of the i-th particle at iteration t, respectively. The parameters $c_{1}$ and $c_{2}$ represent the cognitive and social acceleration coefficients, while w denotes the inertia weight. Furthermore, ${pbest}_{i}^{t}$ refers to the individual best position of particle i, and ${gbest}^{t}$ indicates the global best position attained by the entire swarm.

### 2.3. Shap Principle

SHAP (Shapley Additive Explanations) [33] is a model-agnostic interpretability framework rooted in the Shapley values of cooperative game theory. It is designed to quantify the individual contribution of each feature to specific model predictions. Its core principle is to treat the model’s prediction as the payoff of a “cooperative game,” where the input features serve as the participating players. By calculating the marginal contribution of a feature across all potential subsets and applying a rigorous weighted averaging method, the predictive influence is fairly allocated among all features. The mathematical formulation of the Shapley value is given in Equation (7):

(7) $$y_{i}=\bar{y}+f(x_{i1})+f(x_{i2})+\cdots +f(x_{ik})$$

where $x_{ik}$ is the feature value, $\bar{y}$ is the mean predicted value across the training set, and $y_{i}$ is the specific model output.

### 2.4. Experimental Verification

To validate the engineering applicability and generalization capability of the proposed framework, the optimized model was applied to a dataset of tungsten tailings from a mine in China. First, critical features were identified via SHAP analysis, which provided the basis for a targeted experimental activation program. This program was used to fabricate cement mortar specimens containing activated tailings, followed by 28-day compressive strength testing. Finally, the trained model was used to predict the experimental outcomes, facilitating a systematic evaluation of its performance in practical engineering scenarios.

The main chemical composition of the tungsten tailings is summarized in Table 1. Specimen preparation followed the standard “Test method of cement mortar strength (ISO)” (GB/T 17671-2021) [34]. The mix proportions were designed with a cement-to-sand ratio of 1:3 and a water-to-binder (w/b) ratio of 0.5, with activated tungsten tailings replacing 30% of the cement by mass. After 28 days of standard curing, the compressive strength of the specimens was measured.

### 2.5. Implementation Process

**Figure 1:**
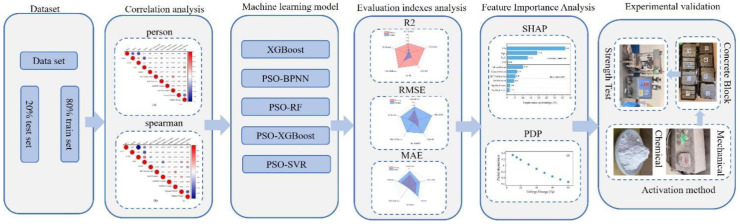
Flow chart.

The technical workflow of this study is illustrated in Figure 1. First, a comprehensive dataset was constructed by integrating compressive strength records from various types of tailings-based cement materials. Prior to model training, the data were normalized to eliminate dimensional discrepancies. To optimize model performance, the PSO algorithm was employed to tune the hyperparameters of the ML models. On this basis, the SHAP method was used to attribute features and identify key influencing factors. In addition, partial dependence plots (PDPs) were used to elucidate the nonlinear relationships and underlying mechanisms between these features and compressive strength. Finally, to validate the model’s generalization performance, an experimental activation program for mine tailings was conducted, guided by the insights from the feature analysis. The model’s predictions were then compared with measured strength values to systematically assess its practical performance.

## 3. Creation and Analysis of Data Sets

### 3.1. Dataset Creation

In this study, a total of 283 data points were compiled from peer-reviewed literature (Table S1). The dataset encompasses experimental results for various activated mine tailings used as supplementary cementitious materials (SCMs). The target (output) variable is the 28-day compressive strength of the tailings-based cement materials. The input features include the chemical composition of the tailings, the activation methods, and the tailings replacement levels.

Mechanical activation is quantified by the specific surface area (SSA) of the tailings. Chemical activation strategies primarily involve alkaline activation and sulfate activation. NaOH, Na_2_SO_4_, and Na_2_SiO_3_ are used as activators for alkali excitation, whereas gypsum serves as the primary activator for sulfate excitation. Incorporating the primary chemical constituents enables the model to account for the mineralogical background of the tailings, thereby enhancing predictive accuracy.

### 3.2. Data Set Processing and Analysis

**Figure 2:**
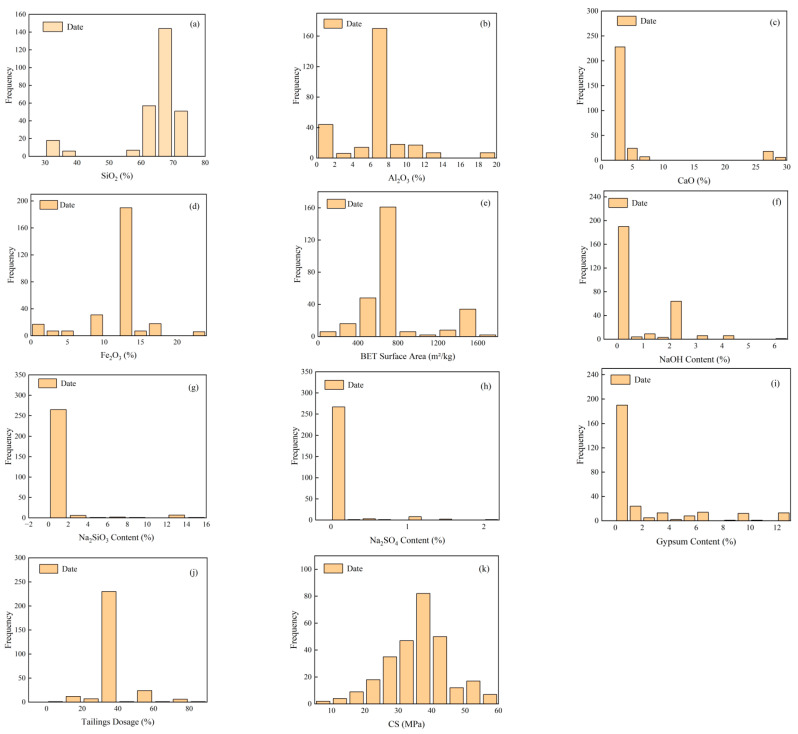
Frequency distribution of characteristic variables and response variables.

In this study, the collected dataset for tailings-based cement materials was meticulously curated and processed. Descriptive statistics—including the minimum, maximum, mean, and median values of the independent and dependent variables—are summarized in Table 2. Additionally, frequency distribution histograms for each variable were constructed to visualize data dispersion, as shown in Figure 2. Figure 2 demonstrates that the dataset has adequate size and balanced coverage for model training.

**Figure 3:**
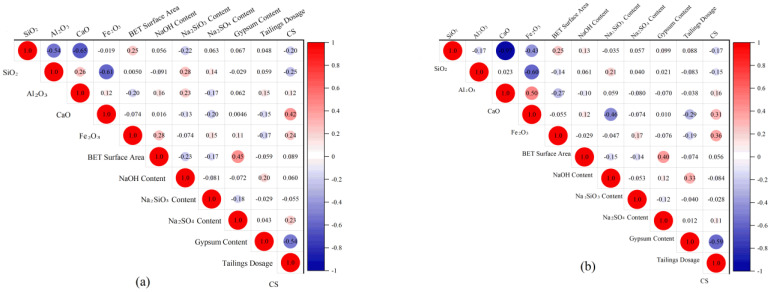
Variable correlations: (**a**) Spearman correlation coefficient matrix, (**b**) Pearson correlation coefficient matrix.

In this study, Pearson and Spearman correlation analyses were used to examine the linear and monotonic relationships within the dataset. Figure 3 shows the correlation coefficient matrices for the input features and between the input features and the output. In the heatmap, red and blue indicate positive and negative correlations, respectively. Coefficients range from −1 to 1, where an absolute value closer to 1 indicates a stronger linear or monotonic dependence. The relationships between the characteristic variables and the compressive strength of tailings concrete were comprehensively analyzed using both Spearman and Pearson correlation analyses. A significant negative correlation was observed between tailings content and compressive strength ($R_{p}$ = −0.59, $R_{S}$ = −0.54).

This trend is primarily attributed to the “dilution effect,” whereby higher tailings replacement levels reduce the relative cement content, thereby limiting the extent of the hydration reaction. The specific surface area ($R_{p}$ = 0.36, $R_{s}$ = 0.24) and gypsum content ($R_{p}$ = 0.11, $R_{s}$ = 0.23) were positively correlated with strength. This positive correlation occurs because mechanical grinding and chemical activation enhance the micro-filling effect and stimulate the secondary (pozzolanic) hydration reaction, thereby improving concrete strength. Furthermore, all correlation coefficients among the input features were below 0.8, indicating the absence of strong multicollinearity. This absence of strong linear redundancy confirms the suitability of the dataset for developing nonlinear machine learning models.

## 4. Results and Discussion

### 4.1. Analysis of Prediction Results

**Figure 4:**
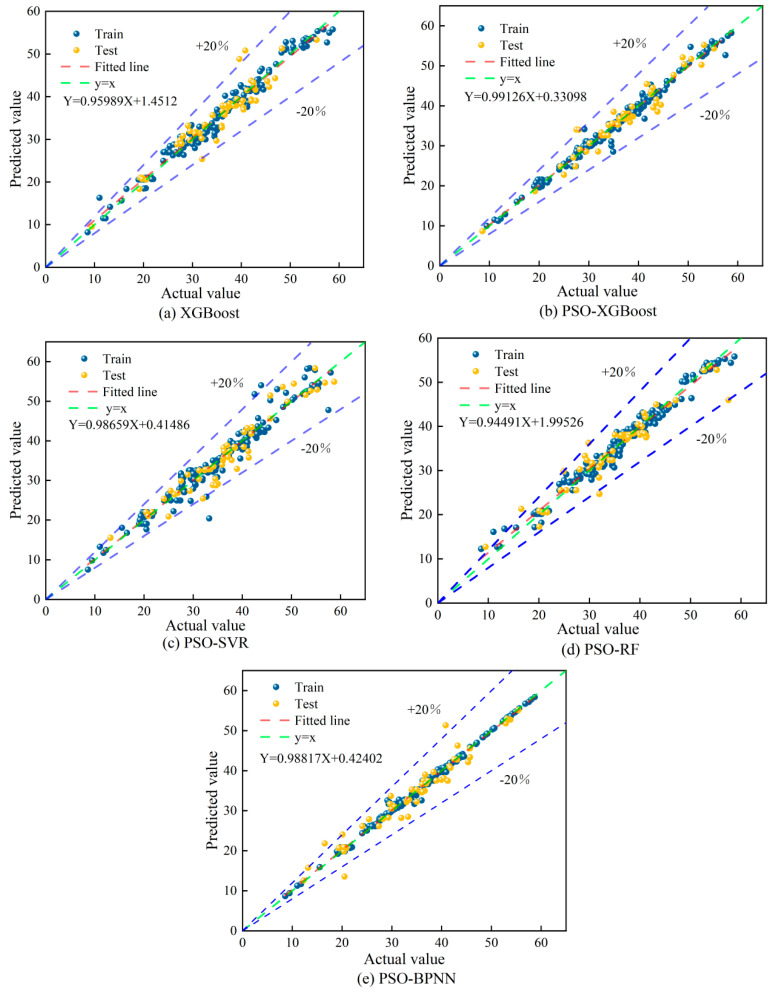
The relationship between the actual value and the predicted compressive strength of each model.

To validate the predictive accuracy of the proposed PSO-XGBoost model, its performance was benchmarked against four alternative models: XGBoost, PSO-BPNN, PSO-SVR, and PSO-RF. The comparative results between the predicted and experimental compressive strengths for all five models are shown in Figure 4. In the figure, the red dotted line represents the model’s fit, the green dashed line represents the identity line (y = x), and the blue dashed lines indicate the ±20% error envelope. The closer the fitting slope of the model on the dataset is to 1, the more accurate the model’s predictions are.

As shown in Figure 4, the test-set slope for the PSO-XGBoost model is 0.9881, and the proportion of predicted values within ±20% of the actual values is significantly higher, indicating more accurate predictions.

**Figure 5:**
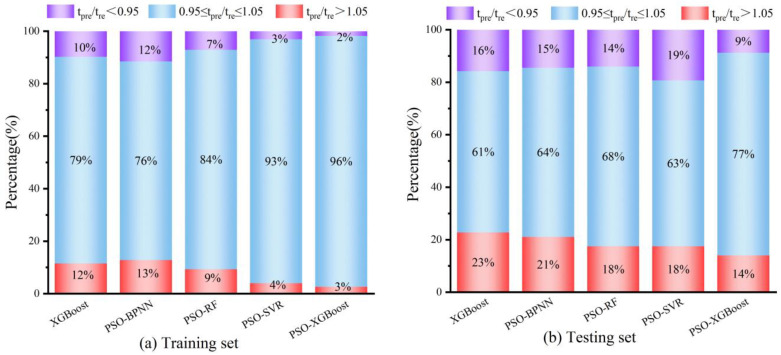
Statistical graphs of tpre/tre values of different models.

As depicted in Figure 5, $t_{pre}$/$t_{re}$ is the ratio of predicted compressive strength to experimental compressive strength. The distribution of the prediction-to-experimental ratio ($t_{pre}$/$t_{re}$) highlights the robustness of the PSO-XGBoost model. Specifically, 96% of the samples in the training set and 77% in the testing set lie within the ±5% error margin (0.95–1.05). This concentration of errors near unity is markedly superior to that of the competing models, confirming the high reliability of the proposed approach.

**Figure 6:**
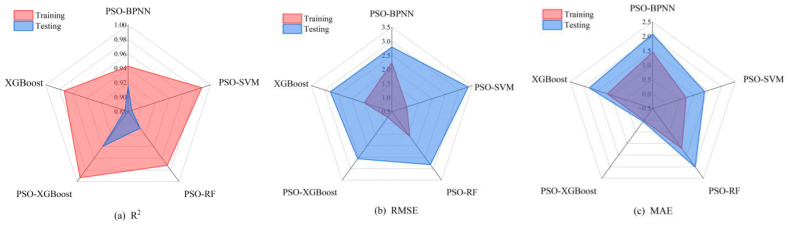
Comparison of evaluation indexes of different prediction models: (**a**) R^2^; (**b**) RMSE; (**c**) MAE.

To systematically assess and compare predictive performance, $R^{2}$, RMSE, and MAE were calculated for both the training and testing phases. On the training set, the PSO-XGBoost model exhibited $R^{2}$ improvements of 5.36%, 0.60%, 2.17%, and 2.07% over PSO-BPNN, PSO-SVM, PSO-RF, and XGBoost, respectively. Correspondingly, RMSE was reduced by 66.5%, 29.2%, 52.5%, and 51.0% for the same model comparisons. MAE decreased by 75.2%, 51.4%, 70.5%, and 68.7%, respectively. These metrics consistently show that the PSO-XGBoost model achieves the highest training accuracy. As shown in Figure 6, the prediction performance of each model on the training set is better than that on the testing set. This observation is consistent with the phenomenon of overfitting, in which models tend to fit the training data more closely than the testing data. Notably, the PSO-XGBoost model exhibited the narrowest performance gap between the training and testing sets. This further substantiates its superior generalization capability and algorithmic stability (Table 3).

In order to evaluate the reliability of the PSO-XGBoost model, the prediction interval method [35] is used to quantify its prediction uncertainty. First, the ratio r between the actual compressive strength and the predicted compressive strength of each sample is calculated, and then a 95% prediction interval is constructed based on the mean and standard deviation of the ratio r, as shown in Equation (8).

(8) PI=[μ−zσ,μ+zσ]

where μ is the average value of the ratio of the actual value to the predicted value, σ is the standard deviation of the ratio of the actual value to the predicted value, and z is the value corresponding to the selected probability level. When the probability is 95%, z is 1.96.

The calculation results show that the prediction interval of PSO-XGBoost model is [0.9264, 1.0729]. The 283 sets of data in the dataset are all within the range of 95% prediction interval, indicating that the PSO-XGBoost model has excellent reliability.

### 4.2. Feature Importance Analysis

**Figure 7:**
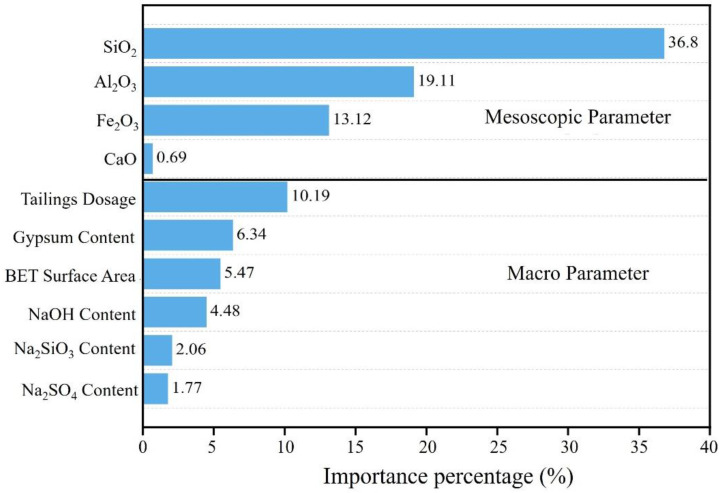
Feature importance graph.

To address the “black-box” nature of machine learning models, the SHAP method was used to elucidate the relationships between the input features and the response variable. As shown in Figure 7, a horizontal bar chart presents the percentage importance of each feature in predicting the 28-day compressive strength of tailings-based cementitious materials. The vertical axis represents the input features, the horizontal axis represents the normalized importance (%), and the sum of the importance values of all features is 100%. The longer the bar, the greater the influence of the feature on the model’s predicted output. The SHAP feature importance analysis results are generally consistent with the previous correlation analysis findings. Among these, SiO_2_, Al_2_O_3_, Fe_2_O_3_, and tailings content emerged as the most critical determinants of the strength of tailings-based cement materials, accounting for a cumulative contribution of 79.22%. Specific surface area (SSA), NaOH dosage, and gypsum content also exhibited significant importance scores. The cumulative contribution of the above seven features is approximately 95.51%, indicating their dominant role in the predictive performance of the PSO-XGBoost model. Mechanical grinding, NaOH content, and gypsum content—among the activation strategies—exert the greatest influence on the compressive strength of activated tailings-based cement materials.

**Figure 8:**
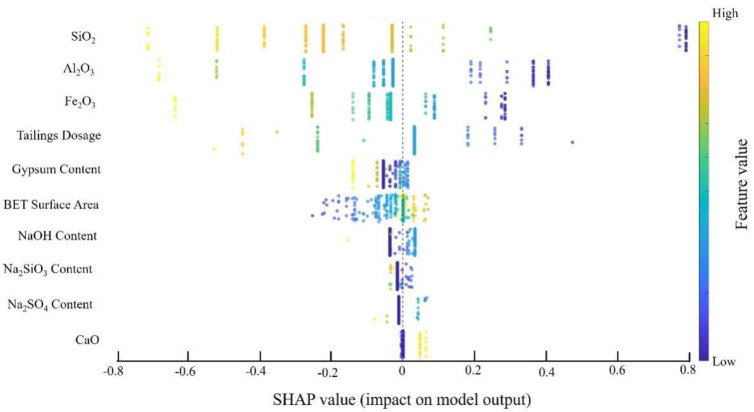
SHAP global interpretation diagram.

Figure 8 provides a global summary plot of the input features, quantifying the direction and magnitude of their influence on strength predictions. As shown in the figure, tailings content is negatively correlated with the compressive strength of activated tailings concrete, where higher values consistently reduce the model’s predicted output. In contrast, specific surface area, NaOH content, and gypsum content are positively correlated with the predicted strength, and increases in these feature values lead to higher predicted strength.

**Figure 9:**
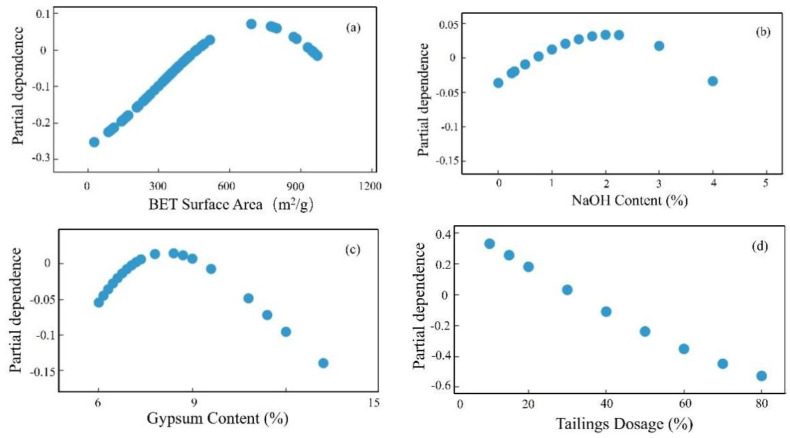
Partial dependence graph.

To further investigate the influence of critical features on compressive strength, partial dependence plot (PDP) analyses were performed for tailings content, specific surface area (SSA), NaOH dosage, and gypsum content (Figure 9). As tailings content increases, the predicted compressive strength exhibits a clear downward trend, confirming a significant negative correlation. This is attributed to the “dilution effect,” whereby higher tailings replacement levels reduce the cement content, thereby decreasing the Ca(OH)_2_ yield and lowering the system’s alkalinity [36]. For specific surface area (SSA) in the range of 0 to 800 m^2^/g, a positive correlation with compressive strength was observed. Exceeding this threshold, however, negatively impacts strength development. This is because moderate mechanical grinding increases the yield of fine particles, enhances the micro-aggregate filling effect, and optimizes the pore structure. Excessive grinding, however, triggers particle agglomeration, hindering the continuous development of mechanical strength [37]. NaOH dosage and gypsum content exhibit similar threshold behavior, with optimal levels of approximately 2% and 9%, respectively. This is because adding appropriate amounts of NaOH and gypsum effectively stimulates the pozzolanic activity of the tailings, promotes secondary hydration reactions, and facilitates the formation of calcium silicate hydrate (C-S-H) gels, thereby fundamentally enhancing matrix strength. Excessive incorporation, however, disrupts the system’s balance and has an adverse effect [38].

### 4.3. Experimental Verification

To validate the predictive performance and generalization capability of the PSO-XGBoost model, an experimental verification phase was conducted. Guided by the SHAP feature importance analysis, optimized operational ranges for mechanical grinding time, NaOH dosage, and gypsum content were established. The ranges of the parameters are as follows: mechanical grinding time, 40–80 min; NaOH content, 1–3%; and gypsum content, 6–12%. Subsequently, a response surface methodology (RSM) experiment was conducted using tailings from a tungsten mine in China. Table 4 presents the correlation between mechanical grinding duration and the resulting specific surface area (SSA) of the tailings, and Table 5 presents the response levels of the experimental factors. The experimental results are presented in Table 6. The optimized PSO-XGBoost model was then used to predict the compressive strength of tailings-based cement materials, with the predictions benchmarked against measured values. The prediction errors remained consistently within ±8.7%, demonstrating the model’s high fidelity and confirming that the PSO-XGBoost model possesses robust generalization capability and reliability for tailings-based cement materials.

## 5. Conclusions

(1) The PSO-XGBoost model demonstrated superior predictive performance over the XGBoost, PSO-SVM, PSO-BPNN, and PSO-RF models, with $R^{2}$ on the test set improving by 6.19%, 6.10%, 2.67%, and 3.41%, respectively. These results show that the PSO-XGBoost model can accurately and reliably predict the compressive strength of tailings-based cement materials.

(2) Feature importance analysis based on the PSO-XGBoost model identified mechanical grinding, NaOH dosage, gypsum content, and tailings replacement level as the primary determinants of compressive strength.

(3) The partial dependence plot (PDP) shows that tailings content is negatively correlated with compressive strength, where higher substitution levels lead to a systematic reduction in strength due to the dilution effect. The specific surface area (0–800 m^2^/kg), NaOH content (0–2%), and gypsum content (0–9%) have a positive effect on the compressive strength of activated tailings concrete within these respective thresholds; beyond them, “over-activation” leads to diminished strength.

(4) Based on the key features identified, tailings from a tungsten mine were tested, and the optimized PSO-XGBoost model was used for prediction. Experimental validation using these tungsten tailings confirmed that the model’s prediction errors remained within an 8.7% margin. This demonstrates that the PSO-XGBoost model has high predictive accuracy and provides theoretical guidance for the sustainable resource utilization of tungsten tailings in the mine.

## Figures and Tables

**Table 1:** Chemical composition of tungsten tailings/wt.%.

SiO_2_	CaO	MgO	Fe_2_O_3_	Al_2_O_3_	SO_3_	Others
64.71	3.69	6.89	12.17	7.59	0.19	4.76

**Table 2:** Statistical table of each parameter.

Input Characteristics and Output Variables	Minimum Value	Maximum Value	Average Value	Median	Standard Deviation
SiO_2_ (%)	30.42	74.89	64.48	67.71	10.34
Al_2_O_3_ (%)	0.76	19.65	7.05	7.59	3.45
CaO (%)	2.1	29.21	5.65	3.69	6.97
Fe_2_O_3_ (%)	1.89	22.14	11.46	12.17	3.77
BET Surface Area (m^2^/g)	45.6	1617	782.43	770	339.45
NaOH Content (%)	0	6	0.68	0	1.08
Na_2_SiO_3_ Content (%)	0	15	0.53	0	2.21
Na_2_SO_4_ Content (%)	0	2	0.054	0	0.24
Gypsum Content (%)	0	12	1.76	0	3.29
Tailings Dosage (%)	0	80	31.77	30	9.81
CS (MPa)	8.57	58.7	35.83	36.6	9.44

**Table 3:** Summary of evaluation results.

Model	Data Set	R^2^	RMSE (MPa)	MAE (MPa)
PSO-BPNN	Train	0.9428	2.24	1.45
	Test	0.9149	2.80	2.08
PSO-SVM	Train	0.9874	1.06	0.72
	Test	0.8854	3.35	1.39
PSO-RF	Train	0.9722	1.57	1.22
	Test	0.9084	2.82	2.01
PSO-XGBoost	Train	0.9933	0.75	0.36
	Test	0.9393	2.56	0.59
XGBoost	Train	0.9731	1.53	1.15
	Test	0.8846	2.79	1.81

**Table 4:** Relationship between mechanical grinding time and specific surface area.

Mechanical grinding time (min)	0	20	40	60	80
Specific surface area (m^2^/g)	420	580	640	770	670

**Table 5:** Levels of response experiment factors.

Factors	Code	Level		
		−1	0	1
Mechanical grinding time (min)	A	40	60	80
NaOH content (%)	B	1	2	3
Gypsum content (%)	C	6	9	12

**Table 6:** Response surface design table.

NO	A	B	C	Test Strength (MPa)	Prediction Strength (MPa)	Relative Error (%)
1	40	1	9	40.74	39.61	2.77
2	80	1	9	40.14	39.62	1.29
3	40	3	9	40.89	39.64	3.05
4	80	3	9	40.62	39.64	2.41
5	40	2	6	40.69	40.44	0.61
6	80	2	6	40.87	40.47	0.98
7	40	2	12	41.22	40.59	1.53
8	80	2	12	40.57	40.73	0.39
9	60	1	6	41.24	42.26	2.47
10	60	3	6	41.53	42.32	1.90
11	60	1	12	41.16	42.37	2.94
12	60	3	12	41.45	42.5	2.53
13	60	2	9	43.75	39.94	8.70
14	60	2	9	41.36	39.94	3.43
15	60	2	9	42.56	39.94	6.16
16	60	2	9	42.3	39.94	5.58
17	60	2	9	43.04	39.94	7.20

## Data Availability

The original contributions presented in this study are included in the article/Supplementary Material. Further inquiries can be directed to the corresponding author.

## Supplementary Materials

The following supporting information can be downloaded at: https://www.mdpi.com/article/10.3390/ma19122557/s1, Table S1: dataset.
